# Health professionals’ experience of nursing home residents’ consumption of alcohol and use of psychotropic drugs

**DOI:** 10.1177/1455072520961890

**Published:** 2020-11-25

**Authors:** Aud Johannessen, Kjerstin Tevik, Knut Engedal, Anne-Sofie Helvik

**Affiliations:** 60512 Vestfold Hospital Trust, Tønsberg, Norway; and University of South-Eastern Norway; 60512 Vestfold Hospital Trust, Tønsberg, Norway; and Norwegian University of Science and Technology (NTNU), Trondheim, Norway; 60512 Vestfold Hospital Trust, Tønsberg, Norway; 60512 Vestfold Hospital Trust, Tønsberg, Norway; Norwegian University of Science and Technology (NTNU), Trondheim, Norway; and St Olav’s University Hospital, Trondheim, Norway

**Keywords:** alcohol, elderly, nursing home, psychotropic drugs, staff

## Abstract

**Background:**

Nursing home (NH) residents are in most cases in older ages and use prescription drugs. As alcohol interacts with many commonly prescribed drugs, NH residents may be more vulnerable to the effects of alcohol.

**Aim:**

To investigate the experiences of health professionals in Norwegian NHs when it comes to residents' alcohol consumption and use of psychotropic drugs, and the facilitation of such use in the NH.

**Method:**

Focus-groups and individual interviews with NH health professionals were performed in 2017 and 2018. The data were analysed using content analysis.

**Findings:**

Two main themes emerged: (1) the balancing of alcohol consumption, and (2) the use of psychotropic drugs. Each of these themes involved reasoning, which revealed that the informants in general had little attention regarding alcohol consumption among residents, and few institutions had policies regarding serving and consumption of alcohol. The informants reported an increased attention regarding use of psychotropic drugs and a tendency towards less use of psychotropic drugs among the residents than before, but few informants reported use of standardised observations tools of symptoms related to prescribing and discontinuation of drugs.

**Conclusion:**

Alcohol policies or procedures related to alcohol consumption were uncommon at the NH that the interviewees of this study represented, and the professionals regarded infrequent serving and consumption of alcohol among the residents as a part of everyday life. In cases when residents frequently consumed alcohol, the professionals used dialogue to underpin the adherent risks and they also tried to control the consumption of the resident in different ways. The interviewees were aware of various side-effects of psychotropic drugs; they were also aware of their effects in combination with alcohol.

In Norway, with a population of five million people, 40,000 nursing home (NH) places (beds) (Statsbudsjett, 2013) offer long- and short-term care, including care in special care units for persons with dementia and rehabilitation. The number of places represents 14% (700,000) of the population aged 65 years or older. Known risk factors for NH admission are poor general medical health, advanced age, dementia, reduced function in activities of daily living (ADL), neuropsychiatric symptoms and having a difficult living situation (De Saint-Hubert et al., 2010; Dramé et al., 2012; Helvik et al., 2015; Roen et al., 2017; Wergeland et al., 2016).

Nursing home residents are very old, have multiple diseases and take on average six to seven drugs on a daily basis (Fog et al., 2019; Jorgensen et al., 2018). Thus, they may be more vulnerable to the effects of alcohol, both with occasional, regular, or elevated consumption compared to healthy older people in general (Menecier-Ossia et al., 2014). Older people are in general more susceptible to the side-effects of alcohol than younger adults, due to age-related changes in metabolism and body composition (Moore et al., 2007). However, older people normally consume less alcohol than their younger peers, and alcohol consumption has been linked to health problems (Holdsworth et al., 2017). Even so, alcohol interacts with many of the commonly prescribed drugs given to frail older people, especially NH residents (Johnson, 2000a).

A recent French study pointed to the importance of having a coherent approach to alcohol consumption in NHs (Menecier-Ossia et al., 2014). A review covering research from 1995 to 1999 reported that few NHs have in-house alcohol policies and that the prevalence of persons with a lifetime history of elevated alcohol consumption in NHs may be extensive, which must be of special concern (Johnson, 2000b). Since alcohol consumption is a normal part of adult social life, some NHs provide alcohol, for example on special occasions aiming to provide enjoyment in life and increase social interaction (Klein & Jess, 2002). Thus, the opinion about alcohol consumption in wider society is also reflected in some NHs, and it may be viewed as a social beverage as well as a psychotropic drug which needs to be controlled. These two views may be seen as competing paradigms, but they are also coexisting realities for NH clinicians and managers (Klein & Jess, 2002). To our knowledge, in Norway there is a lack of information related to NH residents’ alcohol consumption, including access to, consumption of, and consequences of such intake. Moreover, there is a lack of policies regarding the use of alcohol as a social beverage for recreational purposes and alcohol use from a safety perspective in residential care facilities. In Norway, only a few NH units for people with a history of or ongoing severe alcohol and substance abuse are established, and these are located in larger cities (Vossius et al., 2011).

Regarding prescription of psychotropic drugs in NHs, we know that use is high in European countries, including Scandinavia. As many as 42% to 80% of people living in nursing homes are prescribed such drugs (Hosia-Randell & Pitkala, 2005; Huber et al., 2012; Selbaek et al., 2008a). It differs among countries (Feng et al., 2009; Richter et al., 2012) and is higher among residents with dementia or cognitive impairment (Bergh et al., 2011; Gustafsson et al., 2013; Olsson et al., 2010; Selbaek et al., 2008b). Antidepressants are the most commonly used psychotropic drugs among Norwegian NH residents, but benzodiazepines are also frequently used (Kruger et al., 2012; Nygaard et al., 2004; Ruths et al., 2013; Selbaek et al., 2008a). For all psychotropic drugs, except for antipsychotics, an increase in use has been observed over recent decades. A significant decrease in antipsychotics has been seen in recent years (Fog et al., 2019; Ruths et al., 2013).

The use of antipsychotics in Scandinavian NH residents with dementia is among the lowest in Europe (Janus et al., 2016), but it is still considerably high (Helvik et al., 2017), as this group of drugs should not be the first line of treatment for neuropsychiatric symptoms (Jeste et al., 2008; Sink et al., 2005). In demanding clinical situations, antipsychotic drugs may be unavoidable, and their use should be as short as possible, because of the high risk of side-effects (Jeste et al., 2008) and the lack of long-term effects (Sink et al., 2005). Most attention has been given to the side-effects of antipsychotics; even so, there are also serious short- and long-term side-effects with the use of antidepressants and benzodiazepines (Brandt & Leong, 2017; Hartikainen et al., 2007). A recent review from the American Geriatric Society recommend that, in most cases, psychotropic drugs should not be prescribed to older persons (American Geriatrics Society Beers Criterial Update Expert Panel, 2019).

Health professionals working in NHs observe consequences of alcohol and psychotropic drug use, but a study (Iden et al., 2011), shows that health professionals lack knowledge about side-effects. In their daily work, they are in dialogue with the residents and their next of kin regarding consumption of alcohol and use of psychotropic drugs, and eventually they will discuss procedures of use with colleagues and health service agencies. Their attention, reflections and experiences regarding alcohol serving and consumption as well as use of psychotropic drugs may provide information about how to facilitate new routines in NHs.

Thus, it is important to investigate the experiences of health professionals, and in this context, both clinicians and managers who work in NHs, about serving and consumption of alcohol and use of psychotropic drugs. Therefore, we wanted to conduct a study to examine NH staff’s experiences and reflections related to alcohol consumption and use of psychotropic drugs among NH residents, and how to facilitate such use.

## Methods

Qualitative individual interviews and focus-group interviews were performed with NH staff (Berg & Lune, 2012).

### Participants

The informants were included purposively to strive for variation. A total of 19 women and one man, all registered nurses, except for one social educator, were interviewed between December 2017 and October 2018. They were between 24 and 60 years of age and from six counties; their work experience in NH ranged from 6 months to 30 years. Six worked in care units, five had special responsibility for quality development, five were unit leaders and three had other responsibilities in the NH. The informants were contacted either by telephone, letter, or in face-to-face conversations and asked to participate.

#### Focus*-*group interviews

Nine informants participated in focus-group interviews, one consisting of five informants, and one of four informants. Authors AJ and A-SH conducted the focus-group interviews. They were held in a neutral meeting room in a hotel.

#### Individual interviews

Six of the individual interviews were carried out face to face in a neutral meeting room at the participant’s work place or in their private home. Four were carried out by telephone at a time that suited the participants. Further, four of the individual interviews were performed between the first and second focus-group interviews and six after the last focus-group interview. The individual interviews were carried out by AJ.

### Data collection

An interview guide based on thematic questions was applied. It contained three questions focusing on the informants’ experiences with alcohol consumption and the policy in NHs as well as experiences regarding prescriptions and withdrawal from psychotropic drugs (Table 1).

**Table 1.** The main questions asked in the focus-group and individual interviews.

What experiences do you have with alcohol consumption and/or use of psychotropic drugs in the nursing home?
Do you have a policy regarding alcohol consumption in the nursing home?
What are the routines regarding prescription and de-prescription of psychotropic drugs in the nursing home where you work or worked?

Further, depending on the discussions in the focus-group interviews, replies and reflections, the aspects and ideas raised by the informants in the focus-group and the individual interviews led to new questions. New questions were asked and written in field notes and posed again in the next focus-group interview and the individual interviews in order to enrich, elaborate and expand on the given information (Berg & Lune, 2012; Lincoln & Guba, 1990).

A professional typist transcribed the recorded interviews verbatim within two weeks after each interview. Quality control checks of the transcripts were performed by listening to the tapes while reading the interviews by AJ. The interviews lasted 10 to 64 minutes (in total 275 minutes) and were tape recorded.

### Analysis

The transcribed pages of data were analysed using manifest qualitative content analysis (Graneheim & Lundman, 2004). Initially, the transcribed texts were read carefully several times to establish an overall impression. Since no substantial differences as regard to contents or depth were identified between the data collected individually and from focus-group discussions, the analysis was performed similarly. Next “meaning units”, i.e., words and sentences expressing a central meaning, were identified and later systematically condensed without changing the original meaning. At the second stage, the condensed units were labelled with a code stating their content. In the third and final stage, themes and subthemes were created. These consisted of groups of codes according to the themes of the interviews. Special attention was paid to establishing clear differences between and similarities within codes and themes (Graneheim & Lundman, 2004). Authors AJ, and A-SH, had the principal responsibility for the analysis, but the process was continually discussed between the authors.

### Ethics

The study followed the ethical principles outlined in the Declaration of Helsinki (World Medical Association, 2013) and was presented to the Data Protection Service that considered it to be outside the Norwegian Act of Medical and Health Research. Thus, it did not need an approval from the Regional Committees for Medical and Health Research Ethics. Consents from the informants were collected after they had received verbal and written information and before the interviews took place.

## Findings

Two themes related to the NH informants’ opinions and experiences emerged: (1) The balancing of alcohol consumption is needed to improve quality of life and provide good quality of care, and (2) The use of psychotropic drugs, practice and changes related to treatment and care (Table 2). Each theme included two subthemes, which presented different aspects of the informants’ experiences.

**Table 2.** The themes and subthemes that emerged from the structural analysis.

**Themes**	**Subthemes**
The balancing of alcohol consumption is needed to improve quality of life and provide good quality of care	*Alcohol may contribute to pleasure and better quality of life*
	*Alcohol needs to be restricted if alcohol consumption is harmful*
The use of psychotropic drugs, practice and changes related to treatment and care	*Practice and collaboration related to residents’ use of psychotropic drugs*
	*Changes in use of psychotropic drugs as a part of treatment and care*

### Theme 1: The balancing of alcohol consumption is needed to improve quality of life and provide good quality of care

#### Alcohol may contribute to pleasure and better quality of life

The informants indicated that they served or sold alcohol from a trolley in the NH, but only on Fridays and Saturdays, at Christmas or at other festivals where alcoholic beverages are considered normal and a part of Norwegian culture. Serving alcohol, especially wine, on these days was done to improve quality of life. In most cases, it was an underlying assumption that alcohol would give pleasure to the residents. One of the informants said:

I suppose that this practice could easily be changed to rather sell or serve sweets or cakes on the trolley. I do not really know what the residents want. Perhaps it is our way of thinking of what a good weekend or feast should contain in our private life.

Informants also expressed the belief that there should be a balance between offering alcoholic beverages for purchase on the weekend and on special occasions and offering alcohol every day. They did not feel that the majority of the residents wanted to drink alcohol on a daily basis, but some did. The focus-group discussions addressed the residents’ right to make their own decisions about consuming alcohol and whether consumption of alcohol could be harmful to the residents’ health. The attitudes among the staff were that the residents should be given the opportunity to live an active and a normal life as much as possible and not be restricted by the staff or regulations of the NH. One informant said, “We are trying to make the days as good as possible, and not take away what people like”.

The informants also discussed to what extent they should facilitate alcohol consumption in an NH. They concluded that facilitation of alcohol consumption could trigger harmful consumption of alcohol among residents who have had a problematic experience with alcohol earlier in life. It was also suggested that residents’ alcohol consumption could impact other residents’ safety and quality of life. To facilitate the availability of bars in NHs was considered to have both positive and negative effects. It could result in increased quality of life for some of the residents, but also lead to more extensive alcohol consumption. Further, to maintain normal living and strive for residents to have quality of life it was suggested by the informants that one could serve non-alcoholic drinks and wine.

#### Alcohol needs to be restricted if alcohol consumption is harmful

As expressed by the informants, few residents consumed alcohol regularly in NHs. Thus, normally the staff did not focus on the dangers that alcohol consumption could potentially have. Sometimes, the informants said, older people with ongoing alcohol problems are referred to a permanent stay in the NH and stop drinking after the referral. They probably felt safe and stopped drinking. On the other hand, when alcohol consumption was a concern among some residents, the informants expressed that they tried to control their use of alcohol.

Sometimes the staff did not know how much residents drank, because the next of kin or friends of the residents brought it to them. As one staff member said,

We have experienced that the residents behave differently, and we thought that they were ill, but actually they were drunk, because the next day we found an empty wine bottle in the closet. The next of kin had brought wine to the residents. In such cases we lose control over the intake of alcohol.

Another informant said, “We experienced that the next of kin gave alcohol in a children’s cup to a fragile resident”. Others expressed that they did not have a refrigerator in the residents’ rooms, just because they wanted to have control of their intake of beverages.

The informants expressed the importance of exploring the residents’ wishes and having a dialogue with each resident and their next of kin, about the pros and cons of alcohol consumption. This kind of dialogue is important, since the residents or their next of kin may not necessarily know about the dangers of alcohol when health is poor or taken in combination with psychotropic drugs. In such cases, according to one informant,

The next of kin and the residents were told about the dangers, the health personnel’s responsibility for the delivery of psychotropic drugs and the need to have control regarding alcohol intake since it could affect the treatment and health outcome.

The NH’s unit safety programme should include alcohol intake as part of the programme. However, the informants pointed to the fact that guidelines, policies and management focusing on alcohol availability and consumption were lacking, which could lead to different practices and attitudes. One informant stated,

When I work in a group striving for good quality of care, we develop guidelines for the NH. Such guidelines should also regard alcohol consumption. It is important to struggle for implementation at all levels in the organisation, so that it (alcohol availability and use) is linked to health and professional considerations, and not personal values or attitudes.

Different attitudes regarding alcohol consumption in the NH were revealed. In some cases, the staff purchased alcoholic beverages for the residents. Furthermore, it was expressed by the informants that a beer is better than a tranquillizer, so why not purchase a can of beer for the residents instead of giving them a pill? Alcohol was also considered a good treatment. As one of the informants said,

Instead of using tranquillizing psychotropic drugs, Baily’s liquor is served with the coffee, or a glass of red wine is served in the evening so that the residents can have a better night than if they did not get alcohol.

Other informants questioned why they should serve alcohol in the NH. “It is an NH”, as one of them said. Another informant expressed that they have changed their routines over the last three or four years, because they have had some old people with serious problems with alcohol living at the NHs. They changed the routines to allow the residents to have one alcohol unit a day, and the intake was to be in the residents’ own rooms, not in the common living rooms: “One unit a day, I believe, is okay, because many of our residents use psychotropic drugs, both for physical diseases and for psychiatric disorders or on demand”.

### Theme 2: The use of psychotropic drugs, practice and changes related to treatment and care

#### Practice and collaboration related to residents’ use of psychotropic drugs

The informants experienced that older people admitted to NHs are long-term users of psychotropic drugs, and that the dosages have not been changed for years.

As routine during the first week after admittance to an NH, the use of psychotropic drugs is discussed with the new resident and his/her next of kin. Despite this routine, it is often hard to establish a consensus among the health professionals to reduce the use of psychotropic drugs. Further, a few of the informants indicated that some physicians working in NHs did not discontinue drugs often enough. At one NH they did not use psychotropic drugs on a regular basis at all, nor did they use sleeping pills, and as a result, they did not experience the agitation or sleep disturbance associated with discontinuation. As one informant said, “We do not see big differences in behaviour in our NH, because most of the psychotropic drugs have adverse effects”.

Other reasons for ongoing use of psychotropic drugs in NHs were related to lack of human resources and lack of competence among health professionals, as well as to the level of neuropsychiatric symptoms such as severe agitation and aggression among the residents. Some said the NH physicians could find it difficult to evaluate the use of psychotropic drugs, because they received conflicting information from the NH staff. The collaboration between the staff at the NH and the physician was pointed out as important, but also challenging. This collaboration did not work well in all units. According to the informants, one reason for poor collaboration could be that physicians worked part time. In many NHs the consulting physician comes only once a week, whereas the staff observe and reports symptoms and signs of challenging behaviour and possible side-effects of psychotropic drugs among NH residents 24/7. One informant working in an NH unit where the collaboration worked well said,

The physician is employed full time at our NH. When it comes to the focus on drug treatment, we always collaborate with the physician. She discontinues the resident’s use of psychotropic drugs over time and does it systematically.

A whiteboard on the wall was mentioned by one informant as a tool to keep track of actual treatment, including use of psychotropic drugs for each resident. Thus, the need for observation of certain symptoms, effects and side-effects of psychotropic drug prescriptions or discontinuations may be of importance to improve collaboration with the NH physician. As one indicated, “When we started to observe effects and side-effects of psychotropic drugs, we documented and observed the effects every day, evening, and night until the doctor had enough information to be able to make a decision”. Another informant said, “Sometimes we reduce the psychotropic drug use, but now and then we have to put them on again; that happens!”

The narratives revealed some details about the symptoms and effects they observed or reported in connection to prescription or discontinuation of psychotropic drugs.

#### Changes in use of psychotropic drugs as a part of treatment and care

Until recently, the informants did not consider the risk of side-effects of psychotropic drug use. This meant that psychotropic drugs had been used excessively to treat residents with challenging behaviour.

The informants experienced a change of health professionals’ attitudes towards the use of psychotropic drugs in NHs during the past three or four years. Health professionals are today more aware of a non-drug approach to cope with agitation in line with the philosophy of person-centred care, and to offer the residents activities that they enjoy, such as walking outside and playing music. However, the informants gave limited details on when, where and how non-drug interventions could be used.

Further, the informants indicated that the NH staff now spend more time with the residents, and therefore, are more able to solve demanding situations. It was concluded that the use of psychotropic drugs was currently questioned more compared to a few years ago, and that physicians are more prone to ask the NH staff for suggestions of non-drug treatment for challenging behaviour. One of the informants expressed, “When we discuss whether we should use drugs or not, we then try to find the reasons for why these situations come up, and then we investigate a bit further before we eventually solve the situation with pills”.

However, the treatment of agitation with psychotropic drug was still in use in NHs. The informants said this was primarily to protect other residents. As one expressed, “It is important to shield other residents against agitation. That is the main reason for the use of drugs”.

Furthermore, the informants indicated that psychotropic drugs had side-effects, such as drowsiness throughout the day. They expressed a lack of discussion regarding the reasons for such behaviour. They conveyed the need for more knowledge about the use of psychotropic drugs. One person explained, “I need more knowledge and experience. That is why I started to study further”.

Linked to the administration of psychotropic drugs, the use of a multi-dose system came up as an issue of concern. Informants felt that it was difficult to know which drugs had been previously administered, and, therefore, they no longer used the system. One person stated:

You have to cut up the bag and then make the changes, and it is very easy to make errors. It is also hard for us nurses to be updated on medication. Another reason is that it is also quite expensive to use this multi-dose system.

To assure adequate medication treatment, the NHs now had implemented routines for an interdisciplinary medication review once or twice a year. In addition, some NH physicians also asked specialists in geriatrics or geriatric psychiatry for advice in the most complex health situations.

## Discussion

Firstly, our study revealed that there is little systematic attention given regarding alcohol consumption among NH residents. Secondly, different attitudes exist among NH staff towards alcohol availability. Thirdly, there is a lack of policy regarding serving and consumption of alcohol in NHs. Alcohol availability is mainly seen as a desire to please the residents and improve their quality of life. However, in such cases when residents frequently consumed alcohol or when the staff found the consumption to be a health risk, the professionals used dialogue with the residents and tried to control the consumption to ensure appropriate treatment and quality of care.

Our findings show that the use of psychotropic drugs differed between NHs, but there is a tendency towards less use of psychotropic drugs, as this is considered to improve patient safety. Even so, few informants reported that they used standardised tools for observations related to prescribing and discontinuation of drugs. However, in one NH, a regular medication review was introduced as standard procedure, a measure that surely has the potential to improve patient safety and reduce side-effects (Fog et al., 2017).

Further, the findings show that the participants had varied opinions about how and when they should facilitate alcohol consumption in NHs. Alcohol was considered as a cultural symbol that contributed positively to quality of life. Bringing this cultural phenomenon into the NHs was seen as a tool to improve the residents’ lives. In most cases the facilitation of alcohol was based on the underlying assumption that alcohol would give the residents pleasure, but the residents were not necessarily asked what they wanted.

The informants expressed that the residents have the right to decide whether they want to consume alcohol when it involves regular and occasional drinking. The right to decide is in line with the person-centred care approach (Edvardsson et al., 2008; Kitwood, 1997) and the person-directed care concept (Fox et al., 2005). Thus, serving and consuming alcohol in NHs need to be given attention in the perspective of person-centred care and could preferably be included in policy documents and procedures of the NH. These views may be seen as two competing paradigms, but they are coexisting realities for NH clinicians and managers (Klein & Jess, 2002).

However, among those who consumed alcohol regularly, the staff were most focussed on the interaction between consumption of alcohol and the use of drugs. Some informants indicated that the residents do not necessarily know the risks incurred by consuming alcohol regularly. Still, the residents have the right to decide whether they want to consume alcohol regularly or not, but in cases that the staff felt it was risky, appropriate information was given to the resident and next of kin regarding possible side-effects of the combination of drugs and alcohol. The information was included in a dialogue with the involved resident and his/her family. Collaboration gives the patient a voice in relation to treatment and care (Rasmussen & Delmar, 2014).

Next of kin could bring alcohol to the residents, and they might have little knowledge about the harm it could cause when consumed in combination with medication and treatment. The next of kin might also be inclined to do whatever they can to make the best of the situation, but it may not always involve what is best for the resident (Nolan et al., 2004). In such situations a dialogue between the residents, next of kin and the staff was essential, but did not necessarily contribute to a change in the situation. Findings show that the staff tried to balance the decision out of respect to the residents’ right to consume alcohol, but the consumption could contribute to poorer health. Thus, the staff wanted to keep an eye on the consumption of alcohol in NHs to ensure treatment and quality of care.

Furthermore, residents with elevated alcohol consumption could create undesirable situations for other NH residents. There are several considerations to take into account in an NH. In Norway, in some larger cities, NHs have separate units for residents with long-term elevated alcohol consumption (Vossius et al., 2011). In towns and rural municipalities, such challenges need to be solved within the regular units. Thus, NHs could preferably develop in-house alcohol policies including concerns about elevated consumption.

Experience and the practice of the use of psychotropic drugs differed among the NHs. One study (Fog et al., 2017) underlines that practice and use of drugs differs in NHs. During the transition to a NH, many of the older people were users of psychotropic drugs. At the time of admission, the use and the possibility of reducing or discontinuing the use of psychotropic drugs were routinely discussed with the residents and the next of kin. A Norwegian study showed that residents in NHs received three or more psychotropic drugs (Halvorsen et al., 2012). The introduction of a discussion regarding discontinuation of psychotropic drugs is welcome, since polypharmacy and unnecessary usage can lead to side-effects (Fog et al., 2017; Halvorsen et al., 2012).

Our findings also show that there has been a decrease in the use of psychotropic drugs in NHs, especially in antipsychotics. We do not know the reason for this, but maybe the many reports on serious side-effects and the modest effect on challenging behaviour are the reasons (Gulla et al., 2016; Ruths, 2004; Selbaek, 2008). In addition, guidelines and planning documents might have contributed to these changes (HOD, 2008, 2017; Norwegian Directorate of Health, 2009).

Today there is more focus on non-drug treatment, such as person-centred care and social activities. However, the staff said that they lacked competence and knowledge of how to perform such treatment. Lack of competence among the staff has also been the focus of research (Iden et al., 2011).

To ensure the right type and dosage of the medication, one NH included a medication review for each resident every six months and other NHs did this once a year. The latest recommendations from the legislation and the Norwegian guidelines are to have a medication review upon admission to the nursing home and once a year thereafter (HOD, 2015, 2017; Norwegian Directorate of Health, 2018). A Norwegian study found that medication review in NHs resulted in less drug use, especially use of opioids and psychotropic drugs (Fog et al., 2017). Furthermore, to secure patient safety in NHs it is important to observe the effects of the treatment (Bondevik et al., 2017). On the other hand, few informants reported using screening tools or other systematic methods to observe effects and side-effects when prescribing or discontinuing drugs, except for one who used a whiteboard to keep track of the possible effects and side-effects of drugs.

Moreover, the findings show there were also demands linked to administration of medications using multi-dose systems for patient safety. Some NHs no longer used the multi-dose system. Risks related to use of this system are detailed in a previous Norwegian report (Husebø et al., 2017).

### Methodological considerations

The methodological choices in this study were motivated by the lack of previous studies on this topic. Qualitative research in this study consists of methods that are helpful for providing insight into phenomena and subjects that are not well known (Patton, 2002). The present study used a purposive sample of 19 professionals that work in NHs, both as clinicians and managers. The participating professionals worked in different NHs in several regions of Norway; they were all registered nurses (RNs), except for one who was a social worker. They were between 24 and 60 years of age and had been working in this field from 6 months to 30 years. In addition, one professional had experience working with residents who abused alcohol. We believe that this purposive sample helped to validate the results, even if the sample was not purposive regarding gender (Patton, 2002).

Moreover, use of focus-group interviews can contribute to a topic in a broader manner than individual interviews and were valuable to this topic (Kvale & Brinkmann, 2009). We therefore strived to interview nine informants through focus-group discussions, while ten persons were interviewed individually. The reason for not using only focus groups was primarily related to the long distances between some of the participating professionals who worked in an NH. The authors found no clear differences between the data collected individually and in groups.

To enhance the trustworthiness of the data, quotations are presented in the text. In addition, data were analysed and discussed among authors (Lincoln & Guba, 1990). Although our results cannot be generalised in a statistical sense, we argue that they can be transferred to other NHs and countries that focus on alcohol consumption and use of psychotropic drugs among older people in NHs. The results contribute to a better understanding, development and organisation of services for people with elevated alcohol consumption and use of psychotropic drugs and thereby promote health more holistically among residents in NHs.

## Conclusion

The study showed that policies or procedures related to alcohol consumption were uncommon, and the NH professionals saw infrequent serving and consumption of alcohol as a part of life. However, in cases in which NH residents frequently (daily) consumed alcohol, the professionals used dialogue to underpin the risk of such consumption and also tried to control the consumption. The study also revealed that attitudes towards the use of psychotropic drugs is changing. Although NH staff are currently more aware of the side-effects of drugs in combination with alcohol, few reported a systematic approach to observe effects and side-effects related to use, discontinuation, and diverse care strategies.
